# Regulation of the Target of Rapamycin and Other Phosphatidylinositol 3-Kinase-Related Kinases by Membrane Targeting

**DOI:** 10.3390/membranes5040553

**Published:** 2015-09-29

**Authors:** Maristella De Cicco, Munirah S. Abd Rahim, Sonja A. Dames

**Affiliations:** 1 Department of Chemistry, Biomolecular NMR Spectroscopy, Technische Universität München, Lichtenbergstr. 4, Garching 85747, Germany; E-Mails: m.de-cicco@tum.de (M.D.C); munirah.rahim@tum.de (M.S.A.R.); 2 Institute of Structural Biology, Helmholtz Zentrum München, Ingolstädter Landstr. 1, Neuherberg 85764, Germany

**Keywords:** phosphatidylinositol-3 kinase-related kinase, membrane targeting, protein–membrane interaction, signal transduction, mTOR, ATM, ATR, DNA-PKcs, SMG-1, TRRAP

## Abstract

Phosphatidylinositol 3-kinase-related kinases (PIKKs) play vital roles in the regulation of cell growth, proliferation, survival, and consequently metabolism, as well as in the cellular response to stresses such as ionizing radiation or redox changes. In humans six family members are known to date, namely mammalian/mechanistic target of rapamycin (mTOR), ataxia-telangiectasia mutated (ATM), ataxia- and Rad3-related (ATR), DNA-dependent protein kinase catalytic subunit (DNA-PKcs), suppressor of morphogenesis in genitalia-1 (SMG-1), and transformation/transcription domain-associated protein (TRRAP). All fulfill rather diverse functions and most of them have been detected in different cellular compartments including various cellular membranes. It has been suggested that the regulation of the localization of signaling proteins allows for generating a locally specific output. Moreover, spatial partitioning is expected to improve the reliability of biochemical signaling. Since these assumptions may also be true for the regulation of PIKK function, the current knowledge about the regulation of the localization of PIKKs at different cellular (membrane) compartments by a network of interactions is reviewed. Membrane targeting can involve direct lipid-/membrane interactions as well as interactions with membrane-anchored regulatory proteins, such as, for example, small GTPases, or a combination of both.

## 1. Introduction

**Figure 1:**
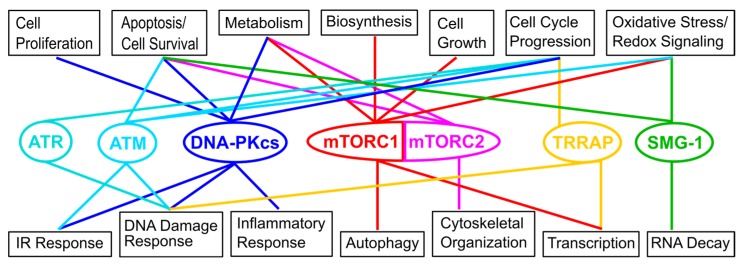
Overview of the six known human PIKKs and their roles in different cellular processes. For the figure information provided in the text and from three reviews has been used [4,5,6]. IR—ionizing radiation.

The family of phosphatidylinositol 3-kinase-related kinases (PIKKs) controls a multitude of cellular signaling branches (Figure 1) in response to different stresses and the availability of nutrients [1,2,3]. In humans six PIKKs have been detected to date: ataxia-telangiectasia mutated (ATM), ataxia- and Rad3-related (ATR), DNA-dependent protein kinase catalytic subunit (DNA-PKcs), mammalian/mechanistic target of rapamycin (mTOR), suppressor of morphogenesis in genitalia-1 (SMG-1), and transformation/transcription domain-associated protein (TRRAP) [2,3]. TRRAP is the only family member not showing catalytic serine/threonine kinase activity [3].

The importance of ATM, ATR, and DNA-PKcs for the DNA damage response (DDR) and signaling in the presence of DNA damage is well known and has been reviewed [3]. The presence of DNA double-strand breaks (DSBs) activates DNA repair by ATM and DNA-PKcs, while ATR responds to the presence of single-stranded DNA (ssDNA) gaps or more generally to DNA replication blocks [2,3]. Moreover, treatment of HT-29 cells with first cis-9, trans-11-conjugated linoleic acid (c9,t11-CLA), substances that can alter the properties of membranes, and then with X-radiation showed delayed DSB repair due to insufficient DNA-PKcs activation [7]. However, these PIKK family members not only regulate DNA repair but also processes such as cell cycle progression and apoptosis (Figure 1) [3,8,9,10]. ATM, but also, as described below, TOR and SMG-1, were further suggested to play a role in redox signaling. The role of ATM in the oxidative stress response and in regulating mitochondrial functions and metabolism has been reviewed [8,11]. DNA-PKcs was further suggested to play a role in the inflammatory response via NF-κB (nuclear factor kappa-light-chain enhancer) [12] and in metabolic gene regulation and the regulation of the homeostasis of cell proliferation [10]. Furthermore, DNA-PKcs is involved in the signaling response to ionizing radiation (IR), which involves phosphorylation of lipid raft proteins [13,14]. Also, ATM has been suggested to be involved in the response to IR [15,16]. Heritable mutations in ATM, ATR, and DNA-PKcs in germline cells lead to ataxia telangiectasia, Seckel syndrome, and severe combined immunodeficiency [2,17,18,19,20,21]. Somatic mutations in ATM have been detected in lymphoma, colon cancer, and lung adenocarcinomas [2,22,23,24]. Inhibition of the interaction of DNA-PKcs with its phosphorylation substrate snail1 has been suggested to be effective in sensitizing cancer cells and reducing metastasis [25].

Mammalian/mechanistic TOR is the best-studied family member and centrally regulates cellular growth and metabolism in response to the nutrient and energy supply and stress conditions such as hypoxia [5,26]. The function of mTOR, like that of ATM, has further been related to redox signaling [6,8,11]. Moreover, the mTOR pathway has been suggested to negatively control ATM [27]. On the other hand, ATM has been suggested to control mTORC1 in response to reactive oxygen species (ROS) and under hypoxic conditions [28,29]. mTOR forms two functionally distinct complexes (mTORC1/2) that intercept different signaling pathways, thereby controlling fundamental cellular processes (Figure 1) [30] including translation, transcription, ribosome biogenesis, lipid metabolism, and autophagy [5,26]. Misregulation of cell growth is involved in the development of metabolic and neurological disorders, tumors, cancer, and pathological changes in organ and body size [5,30,31,32,33]. Only TORC1 is sensitive to the TOR-specific inhibitor rapamycin [30]. The TOR-specific inhibitor rapamycin or derivatives of it, so-called rapalogs, have long been used to suppress the immune response after renal transplantation [33]. Since rapamycin hampers T-cell development, rapalogs are further tested for the treatment of autoimmune diseases such as Parkinson’s disease and multiple sclerosis [34,35]. Dual targeting of mTORC1 and -2 with the inhibitor INK128 was further suggested to be helpful to control HIV (human immunodeficiency virus) in infected patients [36]. The development of new TOR inhibitors is an active field of pharmaceutical research [35,37,38,39].

Suppressor of morphogenesis in genitalia-1 (SMG-1) is well known for its function in nonsense-mediated mRNA decay, but as ATM and TOR plays a further role in oxidative stress as well as cell survival [2,3,40,41,42]. Transformation/transcription domain-associated protein (TRRAP) regulates gene transcription of target genes such as, for example, mitotic checkpoint genes or liver receptors that play a role for lipid metabolism by scaffolding several histone acetyltransferase (HAT) complexes [2,43,44]. As ATM and DNA-PKcs, TRRAP may further be involved in DSB repair processes [45].

**Figure 2:**
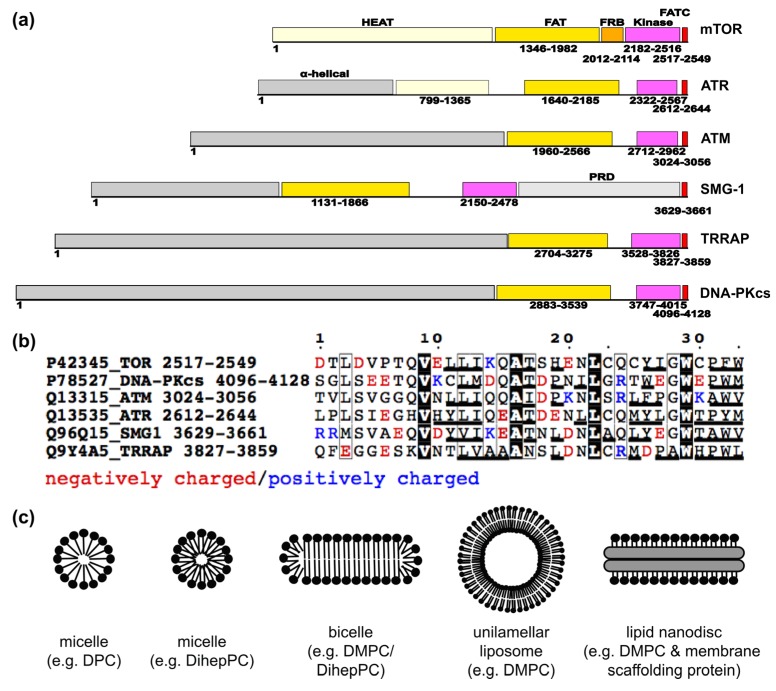
Domain organization of PIKKs, sequence conservation of their FATC domain, and typically employed membrane mimetics. (**a**) The general domain organization of PIKKs, Details are given in the main text; (**b**) sequence alignment of the highly conserved part of the FATC domains (PFAM entry PF02260) of human TOR, DNA-PKcs, ATM, ATR, SMG-1, and TRRAP. The respective Uniprot identification numbers are given at the beginning of each line. Negatively charged residues are colored in red and positively charged ones in blue. Hydrophobic aliphatic and aromatic residues are underlined. The sequence alignment was generated using the program ESPript [58]; (**c**) schematic representations of membrane mimetics typically used for interaction and structural studies, including micelles, bicelles, liposomes of the small unilamellar (SUV) vesicle type, and (protein-) lipid nanodiscs. DPC—dodecylphosphocholine, DihepPC/DMPC—diheptanoyl/dimyristoyl phosphocholine.

**Figure 3:**
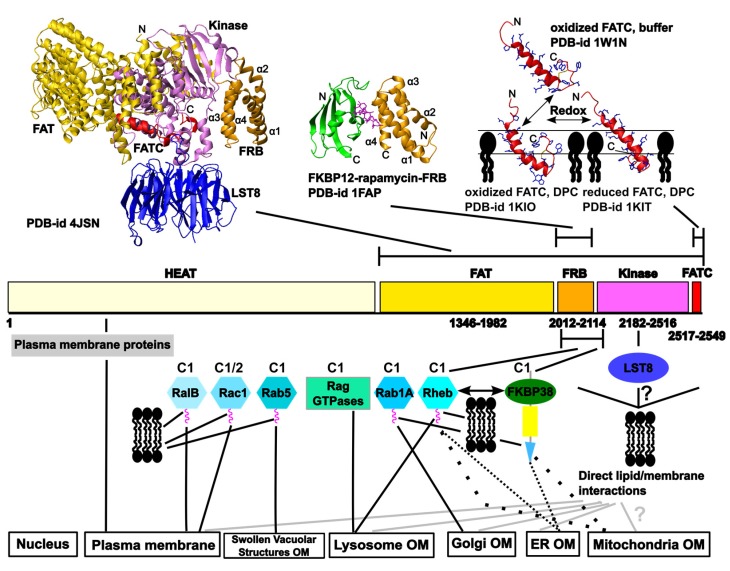
Overview of structural data for TOR and localization information data available for mTOR. Human TOR is 2549 residues long. Details about the domain structure are given in Figure 2a and the main text. The top panel shows the crystal structures of mTOR lacking the HEAT repeat region in complex with LST8 (blue) [50], the FRB domain in complex with rapamycin (magenta) and FKBP12 (FK506-binding protein of 12 kDa, green) [59], and the NMR structures of the oxidized FATC domain in the free [60] *vs.* oxidized and reduced forms in the DPC micelle immersed states [61]; the respective PDB-ids (protein databank identification numbers, [62]) are indicated. The color coding of the TOR domains is the same as in the domain representation below. Below the domain structure, interaction partners that have been [56] suggested to play a role in TOR membrane localization or direct lipid/membrane interactions by TOR domains and the cellular compartments mTOR has localized at are listed. More details can be found in the main text. All structure pictures were generated with the software Molmol [63]. C1 and C2 above the schematic illustrations of some TOR regulatory proteins indicate with which TOR complex they interact. DPC—dodecylphosphocholine; OM—outer membrane.

Although the length of the PIKK amino acid sequences ranges from about 2500 to 4500 residues, they share a highly similar domain structure (Figure 2a). The kinase domain that shows a high homology to lipid kinases is close to the C-terminus [2]. All PIKKs phosphorylate serine and threonine residues in target proteins, except for TRRAP, which shows no catalytic activity [2,3]. All PIKKs contain a further *N*-terminal of the kinase domain a FRAP-ATM-TRRAP (FAT) and a FAT C-terminal (FATC) domain [46]. The N-terminal region with only low sequence homology between different PIKKs was suggested to be mostly composed of α-helical repeat motifs that typically form platforms for protein–protein interactions [46,47,48]. Based on a detailed sequence analysis, the TOR N-terminal region contains mainly HEAT (huntingtin, elongation factor 3, regulatory subunit A of PP2A, TOR) repeats, whereas its FAT domain is mainly composed of tetratricopeptide repeats (TPR) [49]. For the FAT domain, this was further confirmed by a crystal structure of N-terminally truncated mTOR in complex with the protein LST8 (lethal with SEC13 protein 8, Figure 3, upper left), which is a component of both TOR complexes [50]. Based on a recent review about the structural similarities of PIKKs, DNA-PKcs, SMG-1, and TRRAP may also contain an FRB (FKBP12-rapamycin-binding)-like domain between the FAT and the kinase domains [51]. The linker region between the kinase and the FAT C-terminal (FATC) domain has further been referred to as the PIKK regulatory domain (PRD) [2,3,52]. However, this region varies significantly in length and sequence composition between different PIKKs [2,52]. The C-terminal about 35 residues (Figure 2a,b) correspond to the FATC domain (PFAM domain database entry PF02660) [1,46], which is in each family member highly evolutionarily conserved and has been shown to be crucial for the regulation of the kinase domain [2,42,52,53,54,55]. The FATC domains of ATM, DNA-PKcs, and ATR have been proposed to mediate protein–protein interactions [2,55,56]. Those of all human PIKKs may further function as conditional membrane anchors [57].

Targeted membrane localization allows us to spatially separate individual signaling branches of large signaling networks, which is expected to improve the reliability of biochemical signaling processes [64,65]. Mammalian TOR has been localized at the plasma membrane and the outer membranes of the endoplasmic reticulum (ER), Golgi apparatus, mitochondria, and lysosomes as well as in the nucleus and associated with ribosomes [66,67,68,69,70,71,72,73,74,75]. Because of this, the specific output of TOR signaling may depend on its localization, which itself appears to depend on the composition of the two TOR complexes and the signaling state of the cell. In line with the rather diverse set of functions that have also been detected for the other PIKKs, the same may apply for their regulation. Consistent with this, ATM has not only been found to localize in the nucleus but also at cytoplasmic vesicles [15] and at the plasma membrane [76]. Similarly DNA-PKcs has not only been detected in the nucleus but also at lipid rafts [13]. Since regulation of the cellular localization of PIKKs may generally allow a locally specific action in response to IR and other cellular stress factors and signals, we review in the following the significant existing knowledge about the network of interactions mediating the localization of mTOR at different cellular membrane compartments as well as what is known about the membrane-mediating interactions of the other PIKKs.

## 2. Overview of the Network of Interactions Mediating the Localization of the Target of Rapamycin (TOR) at Different Cellular Membranes

Figure 3 illustrates available structural data for mTOR and the current knowledge about the network of interactions mediating and regulating the localization of the two TOR complexes at different cellular membrane compartments. For the FRB domain additional structures alone or in complex with small molecules (e.g., [77,78,79]) and in complex with the FKBP12-like domains of FKBP51 and 52 and rapamycin [80], as well as a structural model for the HEAT repeat region [49], have been published.In the following paragraphs these interactions are described in more detail and complement the review of the localization of TOR in mammalian and yeast cells by Betz and Hall from 2013 [81].

### 2.1. Regulation of TOR Membrane Association by GTPases

#### 2.1.1 TOR Regulators that May Play a Role for the Localization to the Outer Membranes of the Endoplasmic Reticulum (ER), the Golgi Apparatus, and Mitochondria

It has been reported that mTOR localizes to the ER and the Golgi apparatus by localization sequences in the C-terminal HEAT repeat (amino acids 931–1039) and the N-terminal FAT (amino acids 1362–1443) regions [67,71]. Since washing with 4 M urea did not completely remove TOR from the membrane fraction (pellet after centrifugation at 100,000 g = P100), but only a wash with high pH (around 11), it was further suggested that mTOR associates with the ER membrane rather closely [67]. However, the exact interactions that mediate this localization pattern have not been described.

The Ras homolog enriched in the brain (Rheb) and the Rheb like-1 protein (RhebL1) were described many years ago as being able to promote cell growth as a component of the insulin/TOR signaling network [82,83,84]. Later it was shown that Rheb interacts directly with a region encompassing the FRB and the N-terminal part of the kinase domain as well as with LST8 [85]. The tuberous sclerosis complex (TSC), consisting of the proteins hamartin (TSC1) and tuberin (TSC2) as well as TBC1D7, has been identified as a GTPase-activating protein (GAP, GTP = guanosine triphosphate) for Rheb [82,83,86,87]. Rheb is farnesylated within its C-terminal CAAX box, which enables it to localize to endomembranes (e.g., ER, Golgi) and which plays an important role in the interaction with mTOR [88]. Thus, besides the interactions mediated by the abovementioned ER and Golgi localization sequences [61,65], the interaction between TOR and Rheb may also contribute to the rather strong membrane association with these organelles. Localization of mTORC1 at the Golgi apparatus may further be regulated by the GTPase Rab1A [89], which is prenylated and thereby localizes to membranes [90,91]. The interaction with mTORC1 has been suggested to be mediated by raptor (regulatory associated protein of mTOR) and activation of mTORC1 at the Golgi apparatus and at lysosomes (see next section) may represent two distinct amino acid signaling branches [89]. Finally, mTORC1 signaling at the Golgi apparatus may be influenced by polycystin-1 (PC1), since its cytoplasmic tail has been shown to interact with tuberin/TSC1 [92]. The described association of mTORC2 with ribosomes may play a role in its localization to the ER [64,66].

Subcellular fractionation data revealed that mTOR as well as the mTOR–raptor complex and thus mTORC1 can be purified from the mitochondrial fraction [73]. Moreover, the same publication showed that inhibition of mTOR with rapamycin resulted in a strong alteration of the mitochondrial phosphoproteom and suggested that mTOR activity may influence the relative balance between mitochondrial and non-mitochondrial ATP (adenosine triphosphate) sources [73]. It has been suggested that the protein FKBP38 (FK506-binding protein 38), which by a transmembrane domain localizes to mitochondria (Figure 3), is a negative regulator of mTOR in response to growth factor stimulation and nutrient availability [93]. FKBP38 has been suggested to interact by its FKBP12-like domain (FKBP-C) with a region encompassing the FRB and the N-terminal kinase domain region on mTOR (amino acids 1967–2191) [93]. Thus the determined binding site overlaps with one earlier determined for Rheb [85]. In contrast to this result, Bai *et al*. did not see an interaction between Rheb and this region on TOR, but suggested that Rheb interacts in a GTP-dependent manner with FKPB38, thereby preventing binding and thus inactivation of TOR [93]. The model of mTORC1 regulation by FKBP38 proposed by Bai *et al*. has further been challenged by other published work. Wang *et al*. confirmed a preferential binding of FKBP38 to Rheb-GTP and association of mTOR and FKBP38, but could not detect an influence of insulin treatment or serum starvation on the amount of mTOR that got immunoprecipitated by FKBP38 [94]. Uhlenbrock *et al*., on the other hand, had suggested that Rheb copurifies with mTOR but does not interact with FKPB38 [95]. However, they apparently used Rheb protein that was not farnesylated. Based on work by Wang *et al*., a C181S mutant that can no longer be farnesylated is defective in activating TORC1 signaling and cannot bind FKBP38 anymore [94]. Thus further studies are needed to characterize the TOR-Rheb-FKBP38 interaction network and the relevance of membrane association of all binding partners for it. Moreover, it has to be clarified which inputs really regulate it and which (locally) specific outputs this generates. Since FKBP38 has also been shown to interact with the anti-apoptotic proteins Bcl-2 (B-cell lymphoma 2) and Bcl-xL (B-cell lymphoma-extra large), which is regulated by Rheb [96,97], regulation of mTORC1 by FKBP38 and Rheb at mitochondria may link mTORC1 signaling to apoptosis.

#### 2.1.2. The Localization of mTOR Complex 1 (mTORC1) at Lysosomes

The localization and regulation of mTORC1 at the outer membranes of lysosomes/late endosomal structures have been studied in rather great detail and revealed that these processes happen in a highly choreographed manner (reviewed in [37,98,99]). The search for proteins that stimulate mTORC1 in response to amino acid sufficiency resulted in the identification of the Rag (Ras related GTP-binding protein) GTPases that recruit mTORC1 to the lysosome (Figure 3) by interacting with raptor [74,75]. The Rag GTPases (A–D) belong to the Ras (Rat sarcoma) superfamily, but in contrast to other family members contain a long carboxyl-terminal domain, lack a membrane-targeting motif, and can form heterodimers (A/C or B/D) [37,100]. Maximum binding to mTORC1 occurs if A/B are GDP- and B/D GTP-bound [37]. The so-called heterotrimeric ragulator complex acts as a guanine nucleotide exchange factor (GEF) for the Rag A/C or B/D complex and localizes it to the lysosome [101]. The ragulator interacts further with the V-ATPase and is additionally tethered to the lysosomal outer membrane by its lipidated p18 protein subunit [101]. Following recruitment of mTORC1 to the lysosomal membrane, it can be activated by Rheb [74,101]. As was recently revealed, growth factor stimulation leads to phosphatidyl inositol-3 kinase (PI3-K)-dependent activation of PKB/AKT (protein kinase B), which then phosphorylates the TSC complex at multiple sites, thereby resulting in the dissociation of this Rheb-GAP from the lysosome and from Rheb [99]. Accordingly, amino acid signaling to the Rags and growth factor PI3K signaling to Rheb have been suggested to represent parallel, independent inputs on mTORC1 [99].

#### 2.1.3. Further GTPases that May Play a Role in TOR Membrane Targeting

In 2012, the regulation of TOR by small GTPases was shown to include Rheb, Rags, RalA (Ras-related protein A), Rac1 (Ras-related C3 botulinum toxin substrate 1), and some Rab (Ras-related protein) family members [102]. The effects of Rheb, Rab1A, and the Rags on TOR localization and activation are described in the previous two sections. In the following, the roles of additional GTPases for TOR localization and function are summarized.

The RalA-ARF6 (ADP-ribosylation factor 6)-PLD (phospholipase D) complex appears to be involved in the activation of mTORC1 in response to nutrients [102,103] (see also Section 2.2.2). RalB, but not RalA, can interact with mTOR using the same binding region as Rheb [104]. Regarding TOR localization, RalB has been suggested to regulate the serum-induced translocation of mTORC1 to the plasma membrane (Figure 3) [104]. As with most small GTPases, RalB is also lipidated to enable membrane association [105].

The Rho (Ras homologue) family member Rac1 has been reported to regulate both mTORC1 and C2 in response to growth factor stimulation. Rac1 has been suggested to directly interact with TOR, independent of GTP-binding, but dependent on the integrity of the C-terminal region containing the TOR recognition site [106]. In serum-stimulated cells, Rac1 colocalized with TOR not only to perinuclear regions as in serum-starved cells but also at specific membranes, especially the plasma membrane (Figure 3) [106]. Based on sequence similarity, Rac1 is also posttranslationally modified to obtain a membrane anchoring lipid tag (UniProtKB 63000).

Rab5 has been suggested to regulate TORC1 in yeast and mammalian cells and to influence its localization. The authors observed initially mTOR localization to late endosomal/lysosomal compartments; however, overexpression of constitutively active Rab5 appeared to inhibit mTOR by forcing its mislocalization to large swollen vacuolar structures [107]. In yeast, TORC2 has also been suggested to be regulated by Rab-like GTPases [108].

### 2.2. Suggested Direct Lipid/Membrane Interactions of TOR Domains

#### 2.2.1. The FATC Domain of TOR May Function as a Conditional, Redox-Sensitive Membrane Anchor

The structure, redox properties, lipid and membrane interactions, and function of the FATC domain of TOR have been analyzed in detail [53,60,61,109,110,111]. Since it contains two cysteines that are conserved in all organisms, they may form a disulfide bond [60]. The structure of the free oxidized FATC domain (PDB-id 1w1n) consists of an α–helix and a C-terminal hydrophobic disulfide-bonded loop (Figure 3, upper right) [60]. The redox potential determined from a fluorescence-based assay is −0.23 V and thereby similar to the value of glutathione and thus in range, allowing modulation of the redox state by typical cellular redox regulators such as glutathione, thioredoxin, cytochrome c, reactive oxygen species, and other [60]. Complementary *in vivo* mutagenesis studies in yeast showed that replacement of one of the disulfide-bond-forming cysteines lowers the cellular stability of TOR [60]. Based on NMR (nuclear magnetic resonance)-monitored binding studies with different lipids and membrane mimetics (Figure 2c), it has been shown that the FATC domain can further interact with lipids above the critical micelle concentration (CMC), as well as with bicelles [61] and small unilamellar vesicles (SUVs) [110,111]. The structures of the oxidized and reduced DPC (dodecylphosphocholine) micelle-associated forms (PDB-id 2kio, 2kit) are rather similar to that of the free protein (Figure 3, upper right) [61]. However, the α-helix extends further towards the C-terminus, which is more pronounced for the reduced form [61]. Although not restricted by a disulfide bond, the C-terminus of the reduced micelle-associated state also folds back towards the α-helix [61]. The role of different hydrophobic aliphatic and aromatic residues for the affinity for neutral membrane mimetic micelles, bicelles, and SUVs (Figure 2c) has been analyzed by NMR- and CD (circular dichroism)-monitored interaction studies of 15 mutants in total. Based on the results, SUVs at low concentrations appeared overall better suited to detect binding differences between the wild-type and mutant proteins. Moreover, the single and double mutants Y2463A, W2466A, W2466A/W2470A, and Y2463E/W2466E of yeast TOR1 (Y2452A, W2455A, W2455A/W2459A, and Y2452E/W2454E of human TOR) have been suggested to be useful to be incorporated in full-length TOR for *in vivo* functional and localization studies [110]. The publication of a recently determined crystal structure of a human TOR fragment lacking the HEAT region in complex with the protein LST8 (Figure 3, upper left, PDB-id 4JSN) also contained a docking model with a substrate peptide (Supplementary Figure 5 in that publication) [50]. In the therein published structures, the FATC domain interacts with the kinase domain and with one residue with LST8, thereby providing a hydrophobic surface region containing residues Y2452, W2455, and W2459. Thus mutating these residues may influence both substrate binding and the interactions with membranes and membrane-localized TOR regulators such as Rheb, FKBP38, and others [110]. Two studies published in 2000 showed that replacement of W2545 of mTOR with glycine or phenylalanine abolishes mTOR autophosphorylation and mTOR-dependent phosphorylation of eukaryotic initiation factor 4E-binding protein and p70 S6 kinase, and that deletion of the C-terminal tryptophan (W2549) is enough to prevent rapamycin-induced dephosphorylation of p70 S6 kinase [53,112]. The depth of FATC membrane immersion has further been defined based on NMR studies with membrane mimetic micelles doped with spin-labeled stearic acid molecules and complementary MD simulations, and the tilt angle of the single α-helix in neutral and negatively charged lipid bilayers has been estimated from orientated circular dichroism measurements [111]. The combination of these new data with previous structural and interaction data allowed us to publish an experimentally well-founded model for the membrane interactions of the oxidized and reduced TOR FATC domain [111]. Overall the data suggest that the FATC domain of TOR may function as a redox-sensitive membrane anchor that is one component of a network of interactions regulating the localization of the two TOR complexes to different cellular regions [61]. Localized to membranes, the redox state of the FATC domain may further be influenced by lipid oxidation products [61]. Finally, membrane association of the FATC domain does not exclude the possibility of additional interactions with other TOR domains or TOR regulatory proteins [57].

#### 2.2.2. Lipid/ Membrane Interactions by the FKBP-Rapamycin Binding (FRB) Domain

In 2001 it was suggested that the FRB domain may mediate the regulation of TOR by the lipid second messenger phosphatidic acid (PA), which accounts for about 1%–4% of the total lipid content of cellular membranes [113,114]. The generation of PA by phospholipases D1 and 2 (PLD1/2) and by the glycerol-3-phosphate pathway is important for TOR signaling [115,116,117,118]. The activity of the mainly plasma-membrane-localized PLD2 thereby responds to the concentration of diacyl-phosphoinositol-4,5-bisphosphate (PIP45) [114,116]. Moreover, it has been proposed that the interaction of PA with the TOR complexes is competitive with rapamycin and that elevated PLD levels confer rapamycin resistance [116]. NMR studies with a water-soluble PA variant with only C6-fatty acid tails (Dihex-PA) showed that PA induces specific chemical shift changes on a surface region of the FRB domain that is formed by the N-terminal half of α-helix 1 and the C-terminal half of α-helix 4 (Figure 3, upper middle plot) and that overlaps with the binding region of rapamycin-FKBP12 [78]. However, this study did not compare the binding of soluble PA or PA-containing vesicles to that of other negatively-charged soluble lipids or membrane mimetics. Based on later published, more detailed NMR-monitored titrations with water-soluble neutral and negatively-charged short-chain lipids, namely dihexanoyl-PA, -phosphoglycerol (PG), and -phosphocholine (PC) as well as dodecylphosphocholine (DPC) up to ~5 mM, all tested lipids and DPC can interact with the same hydrophobic surface patch [119]. Overall, the interaction with lipids below the critical micelle concentration (CMC) resulted only in small spectral and therefore conformational changes that overall appeared to maintain the fold [119]. In contrast, different membrane-like environments such as neutral or PA-doped negatively-charged micelles and bicelles induced large conformational changes in the FRB domain that largely preserve the α-helical secondary structure content, but appear to disrupt the tertiary structure [119]. Interestingly, SUVs resulted only after longer incubation times in significant spectral changes, either because they were used at significantly lower concentrations as micelles and bicelles or because the interaction may be sensitive to the curvature of the used membrane mimetic [119]. Comparing the effect of neutral and negatively-charged lipids, it has been suggested that the FRB domain has a slightly higher preference for negatively-charged membranes and lipids, but no specific preference for PA or PA-containing membrane mimetics [119]. Thus the FRB domain alone may not be able to mediate the specific effect of PA on TOR signaling. In addition, other negatively-charged lipids or membrane-localized proteins could contribute to this effect. Studies by other groups indicated that PLD-generated PA is required for the interaction of TOR with Raptor in TORC1 and Rictor (rapamycin-insensitive companion of mTOR) in TORC2 [120], whereas PA generated in the glycerol-3-phosphate pathway inhibits TORC2 by destabilizing the TOR–Rictor interaction [118]. These different effects of PA have been explained by structural differences of the PA species generated in both pathways, with only PA species containing palmitate acyl chains (16:0) showing an inhibitory effect on TORC2 [118]. The interaction with PA-containing membrane patches may further be influenced by Raptor in TORC1 and Rictor as well as the presence of different isoforms of Sin1 in mTORC2 [116,121]. This would also be consistent with the suggestion that TORC2 containing Rictor is only sensitive to long-term exposure with rapamycin, because TORC2 has a higher affinity for PLD-generated PA than TORC1 [116].

DPC, which, with its single acyl chain, more resembles a lysolipid, has, as mentioned above, been shown to induce below the CMC chemical shift changes of the FRB domain that are comparable to those induced by the presence of Dihex-PA [119]. Lysolipids are known to strongly influence the local membrane structure [122]. Thus the interactions of either TOR complex with PA-containing membrane patches may further depend on the local membrane composition and the resulting surface properties. Consequently, lysophosphatidic acid (LPA) may not only influence TOR signaling indirectly by being the precursor for the synthesis of PA in the glycerol-3-phosphate pathway [118] or by stimulating PLD1 to produce PA [123], but also by directly influencing TOR–membrane association. Alternatively, another lysolipid could influence the local membrane structure and thereby increase the membrane affinity of the TOR FRB domain. Finally, the interaction of mTORC1/2 with PA-containing membrane patches may additionally depend on the interaction with membrane-associated regulators, for example with phospholipase D2 (PLD2), which not only produces PA but also has been shown to form a complex with TOR/raptor [115] or the GTPases RalA and ARF6 [103].

### 2.3. Further Interactions with Membrane-Localized Proteins

Chlamydomonas TOR and LST8 have also been shown to peripherally associate with internal membranes; however, membrane attachment seemed stronger for TOR than for LST8. Thus it was suggested that LST8 does not anchor TOR to the membrane [124]. However, just as for other β-propeller proteins such as PROPPINs (β-propellers that bind polyphosphoinositides), LST8 membrane association may depend on the surface curvature [125] and thereby mediate additional membrane interactions (Figure 3) that may participate in the regulation of TOR association to specifically composed membrane patches.

In a recently published model for yeast TORC2 plasma membrane association, the PH (pleckstrin homology) domain of Avo1 (adheres voraciously to TOR2 protein 1) interacts with the membrane and occludes the FRB domain, rendering TORC2 insensitive to rapamycin [126]. Moreover, plasma membrane stress has been suggested to induce the association of the membrane-associated Slm1 (synthetic lethal with MSS4 protein 1) and -2 proteins that recognize PI45P by their PH domains, with the TORC2 peripheral subunits Bit61 or Bit2, and Avo2 [127,128]. Rictor is the human orthologue of Avo3, but human orthologues of Avo1, Avo2, and of the Bit and Slm protein are still unknown. However, regarding the localization of human TORC2 to plasma membrane raft regions, syndecan-4 (S4)-dependent recruitment of protein kinase Cα (PKCα) has been suggested to play an important role [129]. S4 can interact with PIP45P by a positively-charged, lysine-rich motif [130].

The C-terminal part of the HEAT region of mTOR has further been suggested to interact with gephyrin, a tubulin-binding protein necessary for the postsynaptic clustering of glycine receptors at the cell membranes of neurons [131]. Moreover, mTOR has further been shown to interact with protein kinase Cδ (PKCδ), a phospholipid-dependent ser/thr kinase [132]. The mTOR–PKCδ complex may further contain the pro-apoptotic and pro-inflammatory transcription factor signal transducer and activator of transcription-1 (STAT1) [133]. The just described interactions may also affect TOR membrane localization; however, it is currently not clear at which cellular membranes and if they affect TORC1, C2, or both.

## 3. The Current Knowledge about Membrane Localization of Other PIKKs

In line with the high abundance of DNA-PKcs in mammalian cells and its roles in DNA repair, the signaling response to IR, metabolic gene regulation, and the regulation of the homeostasis of cell proliferation [10], DNA-PKcs and the two Ku proteins not only localize to DNA damage sites but have been shown to separately localize to lipid rafts of mammalian cells [13]. Because DNA-PKcs appears not to contain a transmembrane domain or to be posttranslationally modified to obtain a GPI or a fatty acid anchor, membrane localization is presumably mediated by protein–protein interactions [13]. DNA-PKcs may further localize to mitochondria by interacting with protein kinase Cδ. PKCδ has been described as proteolytically cleaved and activated at the onset of apoptosis and is known to associate with DNA-PKcs [134]. Moreover, it has been suggested that during the oxidative stress response PKCδ is targeted to mitochondria [135].

Besides DNA repair, ATM plays a role in the oxidative stress response and may act as a linker of genome stability and carbon metabolism [8,11]. Consistent with this, ATM has not only been localized in the nucleus but also at cytoplasmic vesicles [15]. In subsequent work it has been suggested that a portion of ATM localizes to peroxisomes [136]. However, later it was shown that in cells treated with oxidizing hydrogen peroxide, only traces of ATM are found in the peroxisomal light membrane fraction, whereas it was enriched in the mitochondrial heavy membrane fraction [137]. The protein CKIP-1 (casein kinase-2 interaction protein-1), which is involved in muscle differentiation and the regulation of the actin cytoskeleton and cell morphology, has been suggested to mediate localization of ATM at the plasma membrane [138]. Based on additional provided data, the C-terminal region of ATM, including the catalytic and the FATC domain, interacts with CKIP-1 [138]. In response to DNA damage, ATM has further been shown to be acetylated at K3016 in the FATC domain [56,139]. This acetylation depends on the Tip60 histone acetyltransferase, which has been shown to form a stable complex with the FATC domain of ATM [56,139].

ATR has been shown to form a complex with protein phosphatase 5 (PP5) in a genotoxic stress-inducible manner [140]. Since PP5 has been shown to localize to the plasma membrane, which is mediated by its tetratricopeptide repeat (TRP) region interacting with Rac1 [141], ATR may perhaps also localize to membranes when interacting with PP5. In plants, alternative splicing results in a second version of PP5 that contains a transmembrane domain [142]. It has to be analyzed whether the latter is also true for mammalian cells and whether mammalian ATR localizes only to the nucleus or also to cytosolic (membrane) compartments.

In a review about ATM/ATR kinases, the authors mention that they had observed that SMG-1 localizes to the nucleus and the cytoplasm [143]. Otherwise we could not find any information that may point to potential membrane localization at a cytoplasmic compartment. We did not find any publications describing TRRAP’s recruitment to membranes. Regarding the nuclear localization of all PIKKs, the question arises whether interactions with the nuclear membrane may also have to be considered.

Based on NMR- and CD-monitored interaction studies, it has further been shown that the FATC domains of all PIKKs, namely TOR, ATM, ATR, DNA-PKcs, SMG-1, and TRRAP, can interact with membrane mimetics [57,61,110]. In line with differences in the sequence composition (Figure 2b), these data already indicated that the different FATC domains show somewhat different preferences regarding membrane properties such as surface charge and curvature or the packing density of the lipids [57,61]. Whereas the oxidized form of the isolated FATC domain of TOR is well structured (Figure 3, upper right) [61], those of the other PIKKs are rather unstructured and only show a significant increase in α–helical structure in the presence of membrane mimetics [57]. As mentioned above for TOR, membrane interactions of the FATC domain do not exclude it interacting at the same time with proteins, such as, for example, in case of ATM with CKIP-1 at the plasma membrane [138]. In the case of ATM, acetylation in the FATC domain [56,139] may further modulate the interaction with membrane patches or membrane-localized proteins.

## 4. Conclusions Regarding PIKK Activation at Different Cellular Membranes

As described in detail above and summarized in Figure 3, mTOR localizes to many different cellular compartments such as the outer membranes of lysosomes, the Golgi, the ER, mitochondria, and swollen vacuolar structures, as well as to the plasma membrane and the nucleus [66,67,68,69,70,71,72,73,74,75], which seems to be mediated by a network of interactions, the exact nature of which may depend on the cell type as well as the signaling state. Besides the mentioned mTOR localization and activation places, signaling to mTORC1 may further occur from the peroxisome to which Rheb but not the TSC complex or TOR have also been localized [144]; however, this potential activation route remains to be confirmed. Overall, the mTORC1 and C2 signaling network appears to be very complex, which allows it to be finely tuned to a variety of inputs such as growth factors, the availability of amino acids, redox changes/hypoxia, and other factors. Regarding the procedures used for the localization of TOR and its complex partners and regulators, it should, however, be asked how well the methods used can really discriminate between different endosomal membrane compartments that are often connected [145,146,147,148]. In a recent review by Dibble and Cantley, it has been argued that mTORC1 may not really be activated at all the described places since many lysosomal constituents traverse the ER and Golgi complex, Rheb is known to be farnesylated at the ER, and Rab1A regulates trafficking from the ER to the Golgi and potentially other endomembranes [99]. Thus a better understanding of the TOR localization and activation network, as well as those of the other PIKKs, will require further detailed interaction and localization information as well as more structural insights into the individual membrane-localizing interactions and their dependence on the membrane composition, e.g., regarding the presence of typical signaling lipids such as phosphatidic acid (PA) and phosphoinositol phosphate lipids (PIPs) or lipid oxidation products. Recently, the role of protein localization for disease and therapy has been investigated [149]. A future, more detailed analysis of the regulation of PIKK localization and how it is influenced by, for example, specific disease-related mutations may therefore open the route for new therapeutic approaches.
